# Supplementary figures and images for: Modulation of ATP Production Influences Inorganic Polyphosphate Levels in Non-Athletes’ Platelets at the Resting State
Source: Int J Mol Sci. 2022 Sep 25;23(19):11293. doi: 10.3390/ijms231911293 (PMC9570372; doi:10.3390/ijms231911293)

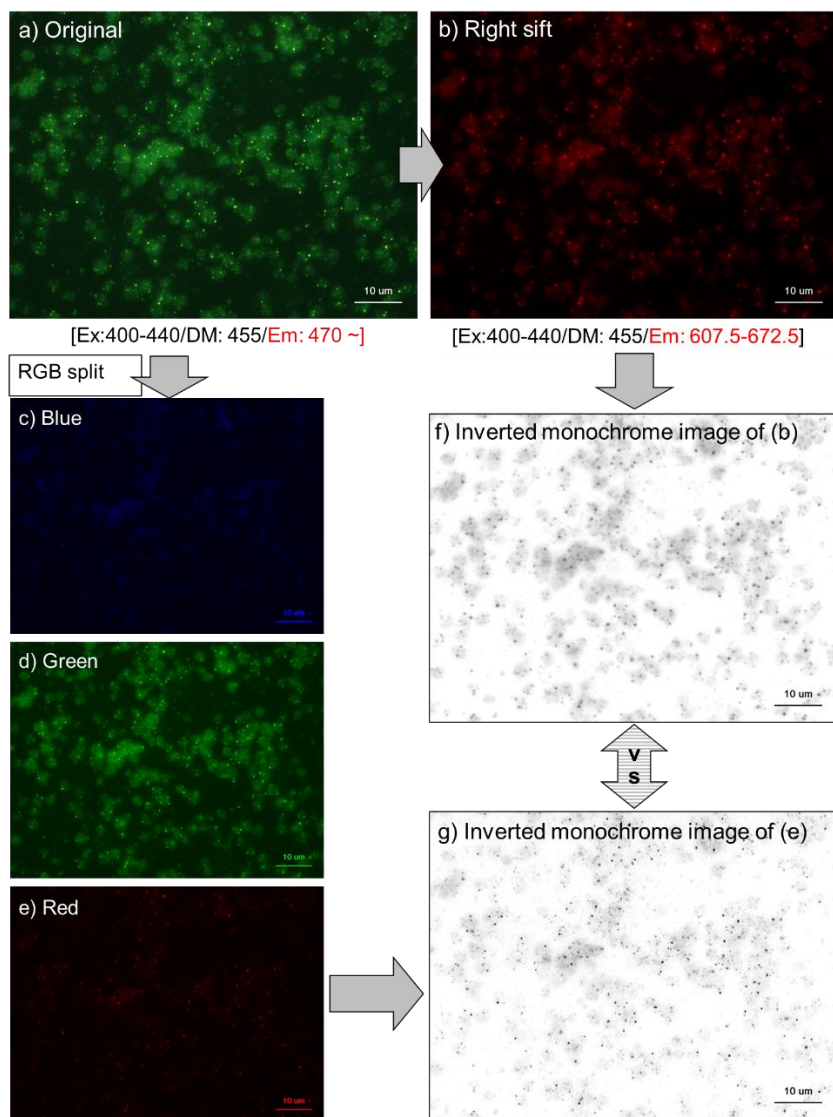

**Figure S1.** Optimization of image analysis of DAPI-reactive microparticles in platelets.

Supplement: Supplementary file 1 [file ijms-23-11293-s001.zip › ijms-1863836-supplementary.pdf]
